# Management of Pyrexia Associated with the Combination of Dabrafenib and Trametinib: Canadian Consensus Statements

**DOI:** 10.3390/curroncol28050304

**Published:** 2021-09-14

**Authors:** Alia Thawer, Wilson H. Miller, Nancy Gregorio, Joël Claveau, Sudha Rajagopal, Kerry J. Savage, Xinni Song, Teresa M. Petrella, on behalf of the Canadian Working Group

**Affiliations:** 1 Department of Pharmacy, Sunnybrook Odette Cancer Centre, Toronto, ON M4N 3M5, Canada; alia.thawer@sunnybrook.ca; 2 Departments of Medicine and Oncology, McGill University, Montreal, QC H3T 1E2, Canada; wilson.miller@mcgill.ca; 3 Princess Margaret Cancer Centre, Toronto, ON M5T 2M9, Canada; Nancy.Gregorio@uhn.ca; 4 Department of Internal Medicine, Dermatology Division, CHU de Québec, Université Laval, Quebec City, QC G1Y 0A1, Canada; joel.claveau@videotron.ca; 5 Credit Valley Hospital, Mississauga, ON L5M 2N1, Canada; srajagopal@cvh.on.ca; 6 Department of Medical Oncology, BC Cancer, The University of British Columbia, Vancouver, BC V5Z 1M9, Canada; ksavage@bccancer.bc.ca; 7 Department of Internal Medicine, The Ottawa Hospital Cancer Centre, University of Ottawa, Ottawa, ON K1H 8L6, Canada; xsong@toh.on.ca; 8 Department of Medical Oncology, Sunnybrook Health Sciences Centre, University of Toronto, Toronto, ON M4N 3M5, Canada

**Keywords:** melanoma, pyrexia, dabrafenib, trametinib

## Abstract

The combination of dabrafenib and trametinib is a well-established treatment for BRAF-mutated melanoma. However, the effectiveness of this approach may be hindered by the development of treatment-related pyrexia syndrome, which occurs in at least 50% of treated patients. Without appropriate intervention, pyrexia syndrome has the potential to worsen and can result in hypotension secondary to dehydration and associated organ-related complications. Furthermore, premature treatment discontinuation may result in a reduction in progression-free and overall survival. Despite existing guidance, there is still a wide variety of therapeutic approaches suggested in the literature for both the definition and management of dabrafenib and trametinib-related pyrexia. This is reflected in the practice variation of its prevention and treatment within and between Canadian cancer centres. A Canadian working group was formed and consensus statements were constructed based on evidence and finalised through a two-round modified Delphi approach. The statements led to the development of a pyrexia treatment algorithm that can easily be applied in routine practice. The Canadian working group consensus statements serve to provide practical guidance for the management of dabrafenib and trametinib-related pyrexia, hopefully leading to reduced discontinuation rates, and ultimately improve patients’ quality of life and cancer-related outcomes.

## 1. Introduction

Inhibition of the mitogen-activated protein kinases (MAPK) signalling pathway through the combined use of BRAF and MEK inhibitors is a well-established approach for treating BRAF-mutant melanoma. The agents lead to a rapid antitumour response and improved survival in both metastatic and adjuvant settings [1,2,3,4,5]. However, almost all patients treated with BRAF and MEK inhibitors develop treatment-related adverse events (AEs) and require special attention to ensure optimal therapeutic benefits without compromising quality of life [6]. While some AEs associated with MAPK inhibitors are class-related, others appear to be agent-specific. Fever with dabrafenib and photosensitivity with vemurafenib are the most common and clinically prominent examples of the latter. Additionally, the majority of AEs tend to occur less frequently with combination therapy, although treatment-related pyrexia is an exception.

In the metastatic setting, the occurrence of pyrexia is notably increased with combination dabrafenib and trametinib (52–71%) versus dabrafenib monotherapy (25–33%) [2,7,8]. Furthermore, in metastatic trials, pyrexia was the most common AE leading to treatment modification, including dose interruption (30–32%), dose reduction (13–14%) and permanent discontinuation (2–3%) [2,9]. The median time to onset of the first febrile episode is approximately 4 weeks, and the median duration is 3–9 days [7,9]. Approximately half of the patients who experienced pyrexia had recurrent episodes [7,8,10]. The incidence of pyrexia is higher when a combination of dabrafenib and trametinib is used in the adjuvant versus the metastatic setting (63% in the COMBI AD trial vs. 52% in COMBI-d and 53% in COMBI-v) [1,2,5]. However, nearly all patients (99%) in the COMBI-AD trial who had pyrexia recovered, with a median time to resolution of 3 days [11]. Furthermore, there was no clinically meaningful decrease in health-related quality of life (EQ-5D-3l scores). To manage pyrexia, 14%, 29% and 69% of patients had dabrafenib withdrawn, dose reduced or interrupted, respectively; for trametinib, the percentages are 9%, 7% and 41% [11]. Phase 3 clinical trials assessing other BRAF/MEK combinations have reported lower rates of any grade pyrexia than with dabrafenib and trametinib (29% with vemurafenib and cobimetinib and 18% with encorafenib and binimetinib) [12,13].

Although the underlying mechanism of combination dabrafenib and trametinib-related pyrexia is unknown, its increased incidence compared to dabrafenib monotherapy, and lack of pyrexia with trametinib monotherapy [14,15], suggests that trametinib influences the dabrafenib-driven pyrexia process [16]. The stimulation of inflammasome activation and interleukin 1 beta production in dendritic cells by BRAF inhibition can lead to pro-inflammatory side effects, including fever [17]. Treatment-related pyrexia does not appear to correlate with any baseline characteristics, and is not predictive of clinical outcome or response to treatment [6,7].

Despite several attempts to standardise the definition and management of dabrafenib and trametinib-related pyrexia [6,7,18], there is still a wide variety of therapeutic approaches suggested in the literature (Table 1) [5,18,19,20,21,22,23]. This is reflected in the various ways dabrafenib and trametinib-related pyrexia is treated within and between Canadian cancer centres (Appendix A, Table A1). Variations in the management of dabrafenib and trametinib-related pyrexia could be related to experience of healthcare professionals with the combination, patient populations, hospital protocols, available resources and access to treatments. The lack of a standardised protocol leads to uncertainty and trial-and-error approaches that can subsequently have negative consequences for patients. Without appropriate intervention, pyrexia syndrome has the potential to worsen and can result in hypotension secondary to dehydration and associated organ-related complications [24]. Furthermore, premature treatment discontinuation due to pyrexia may result in suboptimal oncologic outcomes and a reduction in progression-free and overall survival. 

Due to the unique characteristics of the Canadian healthcare system and access to treatments, there is a need for the development of Canadian-specific recommendations for the prevention and treatment of dabrafenib and trametinib-related pyrexia, rather than adapting existing recommendations [18]. The Australian expert opinion guidelines were, however, used as a valuable resource in the process of developing the Canadian guidelines.

The pyrexia management working group consists of multidisciplinary Canadian healthcare professionals with expertise in the management of melanoma. The group assessed current dabrafenib and trametinib-related pyrexia management approaches and sought to identify proven strategies applied in routine clinical practice. The ultimate goal was to provide Canadian-specific consensus-based recommendations to assist healthcare providers in finding appropriate measures to prevent and treat dabrafenib and trametinib-related pyrexia syndrome.

## 2. Materials and Methods

To reach consensus on the management of treatment-related pyrexia, a modified Delphi process was used [25].

### 2.1. Preliminary Survey

To identify the occurrence of dabrafenib and trametinib-related pyrexia, as well as treatment patterns and challenges associated with its management, a survey was sent to 12 melanoma-treating centres across Canada. The survey collected information about patient volumes, practice distribution, medical and supportive management of first and recurrent pyrexia syndrome and complications related to combination treatment with dabrafenib and trametinib. The results (Appendix A) revealed that despite some similarities, there were significant differences in the management of dabrafenib and trametinib-related pyrexia, which confirmed the need for Canadian consensus statements.

### 2.2. Literature Review

The literature search and review included the following terms: dabrafenib, trametinib, pyrexia, melanoma, BRAF/MEK inhibition and adverse events. The search focused on phase 3 clinical trials with BRAF inhibitors (in particular, protocol-recommendations for the management of dabrafenib and trametinib-related pyrexia (COMBI-AD [5], COMBI-I [22] and COMBI-APlus [23]), relevant product monographs, published review articles [6,7,8] and available treatment guidelines [18]. Several review articles characterise and summarise the incidence of pyrexia with dabrafenib and trametinib [6,7,8], and guidance for its management has been published by two groups [6,18]. The recommendations, along with clinical trial protocols and product monographs, were used as a basis for the development of the Canadian consensus statements.

### 2.3. Panel Members

The steering committee included a multidisciplinary team of 4 healthcare providers with expertise in the management of melanoma: 2 medical oncologists, 1 clinical nurse and 1 pharmacist. The working group included medical oncologists, dermatologists, pharmacists and nurses.

### 2.4. Consensus Process

The modified Delphi methodology adapted by the American Society of Clinical Oncology (ASCO) [25] and Cancer Care Ontario (CCO) [26] was chosen because it provides a formal process for synthesizing expert opinion.

Draft recommendations were developed based on the preliminary survey results, available evidence, guidelines and expert opinion documents. An in-person working group meeting was held on 2 May 2019. During the meeting, the group discussed the available evidence and incidence of dabrafenib and trametinib-related pyrexia reported in clinical trials and routine practice. Available publications and recommendations on the management of pyrexia and the Canadian survey results were used to develop the first draft of the statements.

To obtain consensus on the draft statements, the working group experts participated in a formal consensus process that involved 2 rounds of independent rating of the draft statements and subsequent revisions. For each round, the consensus working group members were asked to rate their level of agreement with each statement on a 5-point Likert scale, ranging from strongly agree to strongly disagree. Those who selected “strongly disagree” or “disagree” were prompted to provide a written explanation of what they disagreed with and why.

Following each round, overall responses and the calculated percent agreement for each statement were used to modify the statements. Percent agreement refers to the number of raters who indicated either “agree” or “strongly agree” divided by the total number of raters for the round. Non-responders are not included in the denominator. The predefined minimum threshold for consensus was ≥75% of raters, indicating agreement with a given statement.

**Figure 1:**
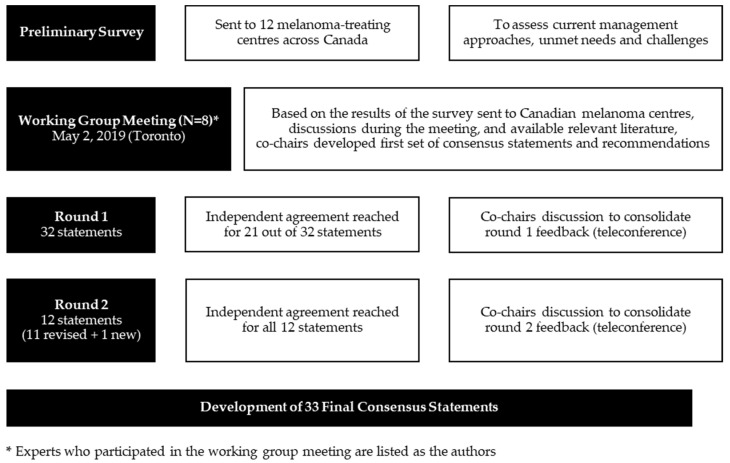
Modified Delphi Consensus Method to Develop Consensus Statements for the Management of Dabrafenib and Trametinib-related Pyrexia Syndrome in Canadian Daily Practice.

The first draft of statements was sent to all working group members for their rating (Figure 1). After the first round, agreement was reached on 21 out of 32 statements. In the second consensus round, the original statements, as well as the modified recommendations, were sent back to the working group for another round of consensus. Statements that did not achieve agreement were either removed or revised according to suggestions from the group members. After the second round, ≥75% agreement was reached for all statements.

## 3. Results

**Figure 2:**
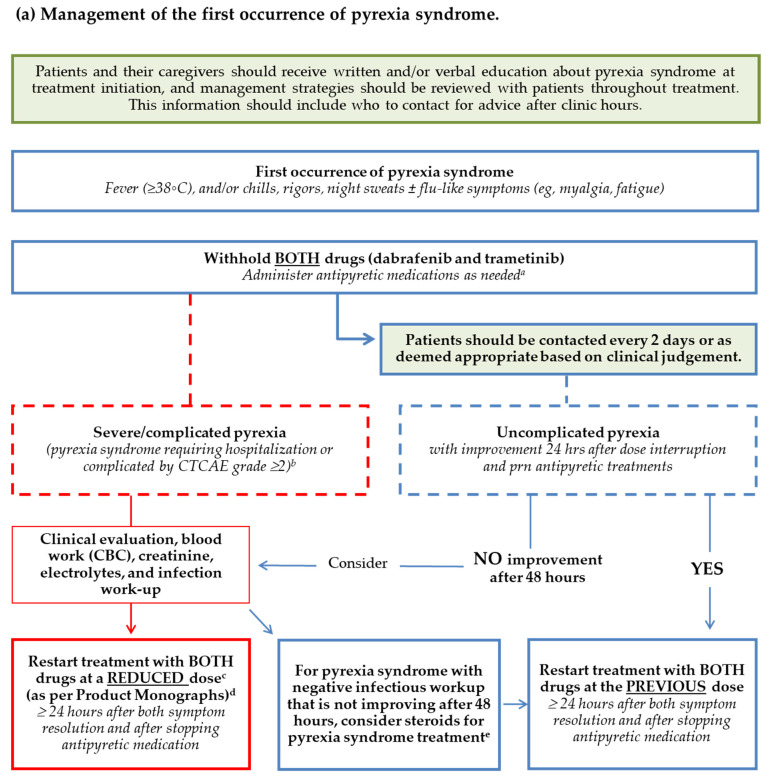

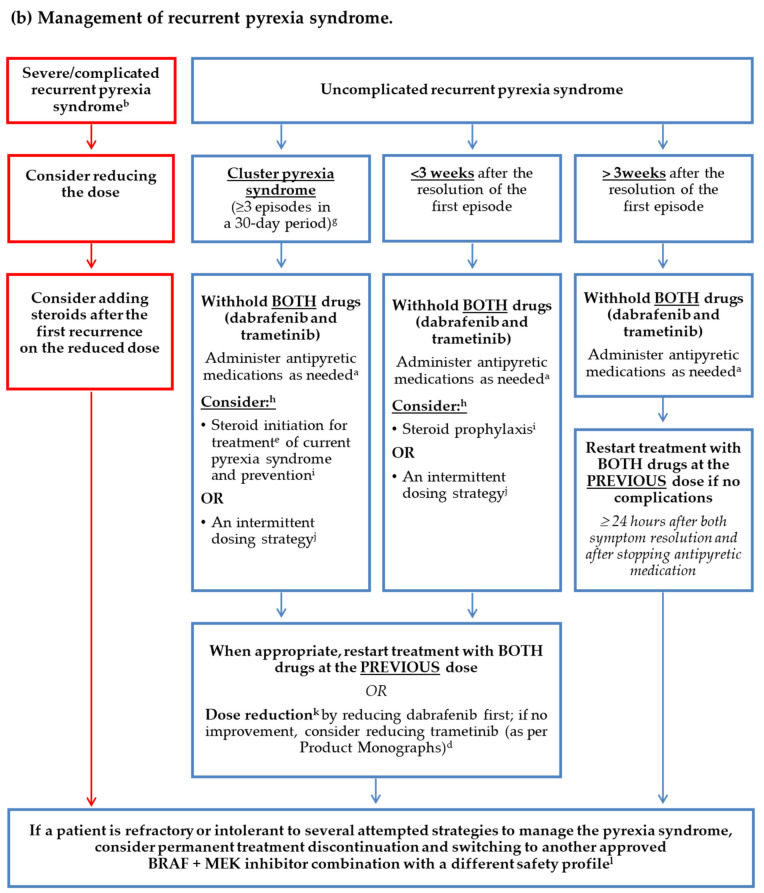
Algorithm based on Canadian Consensus Statements: (**a**) Management of the first occurrence of pyrexia syndrome; (**b**) Management of recurrent pyrexia syndrome. ^a^ Acetaminophen should be given 1 g q 4 to 6 h and not exceeding 4 g per day; Ibuprofen should be given 400 mg q 4 to 6 h and not exceeding 1.2 g per day if self-administering, or 3.2 g per day under close monitoring. ^b^ Severe/complicated pyrexia syndrome is defined as pyrexia syndrome requiring hospitalization or pyrexia syndrome complicated by CTCAE grade 2 or higher dehydration, hypotension, renal dysfunction, confusion or vomiting without another specified cause (e.g., infection). ^c^ Dose escalation of one or both drugs can be considered at a later time if clinically appropriate. ^d^ As per the current product monograph, the recommended dose level reductions for dabrafenib should be as follows: First reduction: 100 mg twice daily (2 × 50 mg twice daily); second reduction: 75 mg twice daily (1 × 75 mg twice daily); third reduction: 50 mg twice daily (1 × 50 mg twice daily); if unable to tolerate 50 mg twice daily: discontinue dabrafenib. As per the current product monograph, the recommended dose level reductions for trametinib should be as follows: First reduction: 1.5 mg once daily; second reduction: 1 mg once daily; if unable to tolerate 1 mg once daily: discontinue trametinib. ^e^ Steroid dosing for treatment of recalcitrant pyrexia syndrome (symptoms not improving with holding dabrafenib and trametinib and antipyretics): prednisone 7.5–25 mg for ≥5 days. ^f^ Recurrent pyrexia syndrome is any subsequent episode of pyrexia syndrome occurring after the resolution of a previous episode and treatment re-initiation. ^g^ Cluster pyrexia syndrome is defined as ≥3 episodes occurring within 30 days. In this case, subsequent episodes recur a few days after the complete resolution of the symptoms associated with the previous episode and treatment re-initiation, leading to ≥3 episodes within a month. ^h^ The decision of whether to use steroids or intermittent dosing should be tailored to the pattern of the occurrence of the pyrexia syndrome, the patient’s needs (i.e., comorbidities) and the setting (i.e., metastatic vs. adjuvant). ^i^ Steroid dosing for prophylaxis: prednisone 7.5–25 mg or dexamethasone 0.5–4 mg daily. Begin titrating downwards if/when the patient has remained pyrexia-free for at least 1 month. ^j^ An intermittent dabrafenib and trametinib dosing strategy includes a short break of 2 to 5 days starting 1 to 2 days prior to an anticipated onset of pyrexia syndrome, followed by treatment at the previous dose upon completion of the treatment break. ^k^ Intermittent dabrafenib and trametinib dosing strategies and/or steroid prophylaxis should be used in patients with frequent recurrent pyrexia episodes before an attempt to reduce the dose of dabrafenib/trametinib. ^l^ When considering the switch, ensure that the novel combination is Health Canada approved for the particular treatment setting (i.e., metastatic vs. adjuvant) and that the patient has access to the new therapy.

The Canadian working group developed a set of consensus statements and a pyrexia-management algorithm (Figure 2) that can easily be applied and followed in routine practice based on the consensus statements. Table 2 provided further recommendations for restarting treatment after the first occurrence of pyrexia syndrome.

A total of 33 consensus statements are divided into the following categories: (1) Definitions; (2) Management of the first occurrence of pyrexia syndrome; (3) Management of recurrent pyrexia syndrome; (4) When to consider steroids/intermittent dosing/and treatment discontinuation; (5) Dosing considerations.

Rationale and additional background information considered when developing the statements are provided where appropriate and applicable.

### 3.1. Consensus Statements: Definitions

**Pyrexia syndrome is defined as fever (≥38 °C), and/or chills, rigors, night sweats ± flu-like symptoms (e.g., myalgia, fatigue).**

The definition of pyrexia and pyrexia syndrome varies between clinical trials and published recommendations (Table 1). While most investigators and working groups agreed that the definition should include a fever ≥38 °C, there are some differences in the inclusion of chills, rigors, night sweats and flu-like symptoms (e.g., myalgia, fatigue). The Canadian working group agreed that pyrexia syndrome could include any combination of chills, rigors and night sweats with or without fever. However, although flu-like symptoms often accompany a fever and/or chills, rigors and night sweats and contribute to pyrexia syndrome severity, due to many other underlying causes, flu-like symptoms in isolation are not sufficient to be considered as pyrexia syndrome.

**Severe/complicated pyrexia syndrome is defined as pyrexia syndrome requiring hospitalization or pyrexia syndrome complicated by Common Terminology Criteria for Adverse Events (CTCAE) grade 2 or higher dehydration, hypotension, renal dysfunction, confusion, vomiting without another specified cause (e.g., infection).**

In the available literature, severe pyrexia is often defined as a fever >40 °C. Based on their experience, the Canadian working group recommends that any pyrexia syndrome requiring hospitalization and/or is accompanied with symptoms classified as grade 2 or higher by the CTCAE definition, is considered severe/complicated and should be further assessed for underlying causes.

**Recurrent pyrexia syndrome is any subsequent episode of pyrexia syndrome occurring after the resolution of a previous episode and treatment re-initiation.**

Although the definition of recurrent pyrexia syndrome is straightforward, its timing in relation to the previous episode is clinically relevant because it impacts therapeutic approaches. Recurrent episode(s) occurring ≥3 weeks after the resolution of the previous episode should be managed differently than pyrexia episode(s) occurring closer together. The recommended approaches are outlined below.

**Cluster pyrexia syndrome is defined as ≥3 episodes occurring within 30 days. In this case, subsequent episodes recur a few days after the complete resolution of the symptoms associated with the previous episode and treatment re-initiation, leading to ≥3 episodes within a month.**

Currently, there is no definition for “cluster pyrexia syndrome” in the literature. However, it is recognised that some patients develop subsequent pyrexia episodes shortly after the resolution of the previous episode and the restart of treatment. As patients who have ≥3 such episodes recurring one after another may need different treatment approaches than those with longer periods between the episodes, it is important to have a clear definition of “cluster pyrexia syndrome” prior to providing recommendations on its management.

### 3.2. Consensus Statements: Management of the First Occurrence of Pyrexia Syndrome

**Patients and their caregivers should receive written and/or verbal education about pyrexia syndrome at treatment initiation, and management strategies should be reviewed with patients throughout treatment. This information should include who to contact for advice after hours.**

Before initiating treatment with dabrafenib and trametinib, patients should be reassured that pyrexia syndrome is generally manageable without requiring permanent discontinuation of treatment. Patients should understand that it is safe and important to temporarily interrupt treatment, and that failure to do so could result in the symptoms persisting and intensifying. Many cancer centres have developed patient counselling strategies and printed materials.

**For the first occurrence of pyrexia syndrome, withhold BOTH drugs (i.e., dabrafenib and trametinib).**

Although product monographs and the COMBI-AD trial protocol suggest attempting to manage pyrexia syndrome by initially withholding only dabrafenib, clinical experience has demonstrated better control of pyrexia syndrome if both drugs are withheld. This approach is recommended in the latest clinical trials with dabrafenib and trametinib (COMBI-I and COMBI-APlus [22,23]), as well as by Australian guidelines [18].

**For the first occurrence of pyrexia syndrome, administer antipyretic medications as needed (dosing considerations are included in Section 3.5).**

The use of acetaminophen or nonsteroidal anti-inflammatory drugs (NSAIDs, e.g., ibuprofen) should be considered to help alleviate symptoms. Alternating between acetaminophen and ibuprofen may help resolve pyrexia syndrome faster, as well as sustain symptom resolution.

**For severe/complicated pyrexia clinical evaluation, blood work (CBC+ diff, creatinine, electrolytes, LFTs), and infection work-up should be completed.**

**For uncomplicated pyrexia, consider clinical evaluation, blood work (CBC + diff, creatinine, electrolytes, LFTs), and infection work-up if after 48 hours there is no improvement despite dose interruption and as needed antipyretic treatments.**

It is important to assess for other potential underlying causes of pyrexia syndrome (e.g., infections) so that these can be treated accordingly. Routine use of antibiotics is not appropriate for patients with pyrexia syndrome. Antibiotics should only be used when the presence of infection has been confirmed or in the setting of grade 3–4 neutropenia. Although rare, all grade neutropenia can occur in 10% of patients and grade 3 or 4 in 3% [19,20]. Oral fluid intake to avoid dehydration should be encouraged. Intravenous fluids should be reserved for patients with poor oral intake or those at high risk for complications.

**During pyrexia-related treatment interruption, patients should be contacted every 2 days, or as deemed appropriate based on clinical judgement.**

Ideally, patients should be contacted every 2 days (in person or over by telephone). However, due to limited resources, this may not always be feasible. In those instances, it is recommended that clinicians use their clinical judgement.

**For uncomplicated pyrexia, restart treatment with BOTH drugs at the previous dose at least 24 h after both symptom resolution, and after stopping antipyretic medication (acetaminophen/ibuprofen).**

Treatment with dabrafenib and trametinib should not be restarted until the patient has been symptom-free for at least 24 h. The Canadian working group also recommends that the patient is off antipyretic medication for at least 24 h as this may “mask” ongoing pyrexia syndrome-related symptoms. See Table 2 for recommendations for restarting treatment after the first occurrence of pyrexia syndrome.

For severe/complicated pyrexia syndrome (requiring hospitalization or complicated by CTCAE grade ≥2 dehydration, hypotension, renal dysfunction, confusion or vomiting without another specified cause (e.g., infection)), restart BOTH drugs at a reduced dose (as per product monographs; see Section 3.5) at least 24 h* after both symptom resolution and after stopping antipyretic medication (acetaminophen/ibuprofen). Dose escalation of one or both drugs can be considered at a later time if clinically appropriate.

* Some working group members indicated that they would wait at least 48 h after the resolution of pyrexia syndrome symptoms before re-initiating treatment.

**For pyrexia syndrome with negative infectious workup that is not improving after 48 h, consider steroids (note: dosing considerations are included in Section 3.5) for pyrexia syndrome treatment.**

Antibiotics should be considered for severe/complicated pyrexia syndrome when an infection is confirmed.

### 3.3. Consensus Statements: Management of Recurrent Pyrexia Syndrome

**Uncomplicated recurrent pyrexia syndrome occurring >3 weeks after the resolution of the first episode: Treat as the first occurrence by withholding BOTH drugs and use of antipyretics as needed (note: dosing considerations are included in Section 3.5). Restart at previous dose if no complications.**

**Uncomplicated recurrent pyrexia syndrome occurring <3 weeks after the resolution of the first episode: Treat by withholding BOTH drugs and the use of antipyretics as needed. In addition, consider: 1. Steroid prophylaxis OR 2. An intermittent dosing strategy (note: dosing considerations are included in Section 3.5) OR 3. Dose reduction when restarting by reducing dabrafenib first; if no improvement, consider reducing trametinib (as per product monographs).**

**Cluster pyrexia syndrome (≥3 episodes in a 30-day period).**

Treat by withholding BOTH drugs and the use of antipyretics as needed. In addition, consider: 1. Steroid initiation for treatment of current pyrexia syndrome and prevention OR 2. An intermittent dosing strategy (note: dosing considerations are included in Section 3.5) OR 3. Dose reduction when restarting by reducing dabrafenib first; if no improvement, consider reducing trametinib (as per product monographs).

**Complicated recurrent pyrexia syndrome: Consider adding steroids after the first recurrence on the reduced dose (note: dosing considerations are included in Section 3.5).**

When managing recurrent pyrexia syndrome, it is important to take into consideration the timing of its recurrence (i.e., < or >3 weeks since the previous episode), frequency (i.e., ≥3 episodes in 30 days) and associated complications. For uncomplicated recurrent pyrexia syndrome, the Canadian working group recommends steroid prophylaxis prior to attempting intermittent dosing.

### 3.4. Consensus Statements: When to Consider Steroids/Intermittent Dosing/and Treatment Discontinuation

**Consider steroid prophylaxis in cases of cluster pyrexia syndrome (≥3 episodes in a 30-day period; note: dosing considerations are included in Section 3.5).**

**Consider steroid prophylaxis in cases of frequent recurrent episodes (note: type of steroid and dosing considerations are included in Section 3.5).**

Data on steroid use in patients with dabrafenib and trametinib-related pyrexia syndrome are limited. The Australian guidelines recommend corticosteroids as a prophylactic measure with tapering if/when the patient has remained pyrexia-free for at least one month [18].

**Current data support consideration of the use of intermittent dabrafenib and trametinib dosing to manage treatment-related pyrexia (note: type of steroid and dosing considerations are included in Section 3.5) [7,17,18,19,20].**

**The decision of whether to use steroids or intermittent dosing should be tailored to the pattern of the occurrence of the pyrexia syndrome, the patient’s needs (i.e., comorbidities) and the setting (i.e., metastatic vs. adjuvant) (note: type of steroid and dosing considerations are included in Section 3.5).**

**Intermittent dabrafenib and trametinib dosing strategies and/or steroid prophylaxis should be used in patients with frequent recurrent pyrexia episodes before an attempt to reduce the dose of dabrafenib/trametinib (note: dosing considerations are included in Section 3.5).**

Dose reduction, according to a single centre experience, may not be as effective as other strategies (steroids or treatment interruptions) in the management of dabrafenib and trametinib-related pyrexia [12].

**Consider permanent treatment discontinuation in case of serious AE(s).**

**Consider permanent treatment discontinuation if the patient is refractory to dose reduction and steroids.**

**Consider permanent treatment discontinuation if the patient did not tolerate any suggested strategy.**

**If a patient is refractory or intolerant to several attempted strategies to manage the pyrexia syndrome, consider switching to another approved BRAF + MEK inhibitor combination with a different safety profile (i.e., encorafenib and binimetinib or vemurafenib and cobimetinib). When considering the switch, ensure that the novel combination is Health Canada approved for the particular treatment setting (i.e., metastatic vs. adjuvant) and that the patient has access to the new therapy.**

The availability of targeted therapies with different tolerability profiles provides additional options for the management of BRAF-mutated melanoma. However, consideration should be given to side effects associated with other BRAF–MEK combinations as well as patient comorbidities and patient preferences.

### 3.5. Consensus Statements: Dosing Considerations

**Acetaminophen should be given 1 g q 4 to 6 h and not exceeding 4 g per day.**

**Ibuprofen should be given 400 mg q 4 to 6 h and not exceeding 1.2 g per day if self-administering, or 3.2 g per day under close monitoring.**

Lower doses should be considered if alternating between acetaminophen and ibuprofen, depending on the severity and duration of pyrexia syndrome.

**Steroid dosing for prophylaxis: prednisone PO 7.5–25 mg or dexamethasone 0.5–4 mg PO daily. Begin titrating downwards if/when the patient has remained pyrexia-free for at least 1 month.**

Clinicians should use their clinical judgement and tailor the dose and duration of treatment with steroids, according to the severity and timing of pyrexia syndrome (i.e., first vs. subsequent occurrence) and other patient- and disease-related characteristics.

The recommendation to continue for at least 1 month is also recommended by the Australian group [18].

**Steroid dosing for treatment of recalcitrant pyrexia syndrome (symptoms not improving with holding dabrafenib and trametinib and antipyretics): Prednisone 7.5–25 mg for ≥5 days.**

**An intermittent dabrafenib and trametinib dosing strategy includes a short break of 2 to 5 days starting 1 to 2 days prior to an anticipated onset of pyrexia syndrome, followed by treatment at the previous dose upon completion of the treatment break.**

The supporting evidence for the intermittent use of dabrafenib and trametinib is somewhat controversial and remains a topic of discussion at scientific meetings. Proactive intermittent dosing was not allowed in the COMBI-I [22] or COMBI-APlus [23] trials with dabrafenib and trametinib. According to results from the SWOG S1320 trial, presented at the 2021 virtual American Association for Cancer Research (AACR) meeting, continuous dosing with the BRAF and MEK inhibitors dabrafenib and trametinib yields superior progression-free survival (PFS) compared with intermittent dosing [27]. Intermittent dosing in this trial included a 3-week-off, 5-week-on schedule.

On the other hand, the Australian guidelines recommend an intermittent dosing strategy at a full dose that includes continuous treatment for 12 days followed by a 2-day break for patients experiencing pyrexia syndrome every 2–3 weeks [18].

Some experts suggest that intermittent dosing (short “drug holidays” of ~2–5 days) is an effective management strategy where a full dose can be maintained. This is unlikely to impact efficacy and is preferable to dose reduction [7].

The recommendation for intermittent dosing is in theory supported by the pharmacokinetic properties of the drugs. The terminal half-life of dabrafenib is 10 h [17,20]. Based on the induction half-life (67 h), a steady-state should be achieved within 14 days of dosing [17,20]. The mean terminal half-life of trametinib is 5 days. The steady-state is estimated to be achieved within 20 days following the administration of 2 mg once daily [19].

**As per the current product monograph, the recommended dose level reductions for dabrafenib should be as follows:**

- **First reduction: 100 mg twice daily (2 × 50 mg twice daily);**
- **Second reduction: 75 mg twice daily (1 × 75 mg twice daily);**
- **Third reduction: 50 mg twice daily (1 × 50 mg twice daily);**
- **If unable to tolerate 50 mg twice daily: discontinue dabrafenib.**

**As per the current product monograph, the recommended dose level reductions for trametinib should be as follows:**

- **First reduction: 1.5 mg once daily;**
- **Second reduction: 1 mg once daily;**
- **If unable to tolerate 1 mg once daily: discontinue trametinib.**

The provided dosing recommendations are based on Canadian working group members’ experience, published evidence and product recommendations. However, as dosing of antipyretic drugs and/or steroids may depend on other patient- and disease-related factors, including comorbidities and concomitant therapies, the Canadian Working group encourages clinical judgement and discretion.

## 4. Discussion

Management of dabrafenib and trametinib-related pyrexia syndrome presents significant challenges to treating clinicians, mainly due to the lack of standardised treatment recommendations. The discrepancies and differences in the recommended approaches are apparent at multiple levels, from the clinical trial protocols and the product monographs to hospital protocols and individual practices. While the dabrafenib and trametinib product monographs recommend withholding only dabrafenib until the resolution of pyrexia symptoms [19,20], the most recent trials recommend withholding both drugs [22,23]. The initial survey of 12 Canadian melanoma-treating centres revealed variations in the duration of treatment interruptions, use and dosing of antipyretic therapies, prophylactic approaches, etc.

The Canadian working group used a three-step modified Delphi method to develop the consensus statements and recommendations. The statements are in line with previously published recommendations by the Australian group [18], but provide a more stepwise approach and direction. In addition, the Canadian working group further defined recurrent pyrexia syndrome and intermittent dosing, both of which are commonly seen in clinical practice but poorly defined in the literature. The consensus statements also provide suggestions regarding the dosing of antipyretics and steroids. While dosing of antipyretics and steroids should be left to the clinical judgement of treating clinicians, the reassurance that it is appropriate to reach for higher doses, if needed, could provide clinicians with additional comfort when making treatment-related decisions.

Two studies have assessed the use of an adaptive pyrexia algorithm and have shown improvement in pyrexia-related outcomes without compromising survival outcomes [28,29]. A pyrexia treatment algorithm has been developed to provide practical guidance for the management of pyrexia syndrome, with the intent of reducing discontinuation rates, and ultimately improve patients’ quality of life and cancer-related outcomes. These data supplement and compliment the Canadian consensus statements.

Although based on available scientific evidence, a limitation of the Canadian pyrexia management consensus statements is that they might unintentionally reflect the expertise and opinions of the working group members. Thus, the algorithm for the management of dabrafenib and trametinib-related pyrexia should be considered as a flexible tool that is based on the best available scientific evidence and clinical information. It reflects the consensus of experts in the field while allowing clinicians to use their individual judgement in managing their patients.

## 5. Conclusions

The combination of dabrafenib and trametinib is an important therapy for patients with BRAF-mutant melanoma. To streamline management of pyrexia syndrome, a common AE associated with this combination, and help prevent unnecessary treatment cessations, the Canadian working group has provided a set of consensus statements and recommendations using a modified Delphi approach along with the proposed pyrexia syndrome management algorithm. Appropriate management of dabrafenib and trametinib-related pyrexia syndrome could improve patient quality of life, prevent unnecessary switching between therapies and help ensure optimal treatment outcomes.

## Figures and Tables

**Table 1:** Different definition and recommendations for the management of pyrexia syndrome.

Sources	^Pr^TAFINLAR^®^ (dabrafenib) Product Monograph [20]	COMBI AD [5,21]	COMBI I [22]	COMBI-APlus [23]	Australian Guidelines [18]
Definition of pyrexia/pyrexia syndrome	Pyrexia definition is not explicitly stated. Guidance for fever ≥ 38.5 °C	Pyrexia defined as body temperature ≥ 38 °C	Pyrexia syndrome: Treatment related fever (≥38 °C) or Chills/rigors/night sweats or Flu-like symptoms	Pyrexia: Fever occurring while on study treatment (≥38 °C) Pyrexia syndrome: one or more of the following symptoms: Chills/rigors/night sweats Flu-like symptoms	Pyrexia syndrome: Presence of any of the following symptoms: Fever ≥ 38 °C Chills, rigors, night sweats, flu-like symptoms Flu-like symptoms
Definition of severe pyrexia	Fever > 40 °C or any fever with complications: Severe rigors or chills, dehydration, hypotension or renal failure in the absence of another cause (e.g., infection)	Fever >40 °C or associated with rigors, severe chills, dehydration or hypotension, serum creatinine and other evidence of renal dysfunction			Fever that does not improve within 24 h Confusion Localizing symptoms Vomiting and/or dehydration
Infectious Workup	Any fever occurrence			Laboratory work-up and clinical evaluation for infection for patients with pyrexia not resolving within 24 h	Fever that does not improve within 24 h Confusion Localizing symptoms Vomiting and/or dehydration ANY symptoms present at 5 days
Management of first occurrence of pyrexia syndrome					
Treatment interruption	Interrupt dabrafenib if uncomplicated fever 38.5–40 °C; Continue trametinib	Interrupt dabrafenib if uncomplicated fever ≥38 °C; Continue trametinib	Interrupt both drugs for pyrexia syndrome	Interrupt both drugs if uncomplicated fever ≥38 °C.	Interrupt both drugs for pyrexia syndrome
Restart	Restart dabrafenib at same or reduced dose once fever resolves	Restart dabrafenib at resolution of fever at same dose	Restart both drugs at same dose once symptom free for at least 24 h	Restart both drugs at same dose once symptom free for at least 24 h	Restart both drugs at same dose once symptom free for at least 24 h
Management of subsequent occurrence of pyrexia syndrome					
Dose reduction	Dabrafenib only	Dabrafenib in patients experiencing pyrexia not controlled by antipyretics or associated with rigors, severe chills, dehydration, hypotension or renal insufficiency	Can be considered if interruptions unmanageable *	Can be considered if recurrent pyrexia cannot be managed with interruption or prophylactic steroids, dose reduction is required *	If intermittent dosing and corticosteroid prophylaxis fail, consider dose reduction (only as a last resort)
Steroids	If antipyreticsineffective in treating fever	Recommended for treatment of pyrexia not controlled by antipyretics or associated with rigors, severe chills, dehydration, hypotension or renal insufficiency AND any second or subsequent occurrence	For fever treatment where antipyretics insufficient As clinically indicated for recalcitrant pyrexia	Recommended as treatment for recurrent pyrexia that cannot be managed with dose interruptions and antipyretic treatments and for pyrexia associated with complications. Consider as prophylaxis to prevent further episodes of pyrexia in those with recurrent pyrexia events	Recurrent or severe pyrexia syndrome as prophylaxis
Intermittent dosing	Not mentioned	Not mentioned	Not allowed	Not allowed	Recurrent or severe pyrexia syndrome

^Pr^TAFINLAR^®^ (Novartis Pharmaceuticals Canada Inc., 385 Bouchard Blvd., Dorval, QC, Canada); *: Not drug specific.

**Table 2:** Recommendations for restarting treatment after the first occurrence of pyrexia syndrome.

Pyrexia Syndrome Severity	Treatment Restart
Uncomplicated pyrexia with improvement 24 h after dose interruption and antipyretic treatments (as needed)	Restart treatment with BOTH drugs at the PREVIOUS dose ≥24 h after both symptom resolution and after stopping antipyretic medication
Uncomplicated pyrexia not improving after 48 h and negative infectious workup	Consider steroids for pyrexia syndrome treatment Restart treatment with BOTH drugs at the PREVIOUS dose
Severe/complicated pyrexia (pyrexia syndrome requiring hospitalization or complicated by CTCAE grade ≥2)	Restart treatment with BOTH drugs at a REDUCED dose (as per product monographs) ≥24 h after both symptom resolution and after stopping antipyretic medication

## Data Availability

All data are presented.

## Appendix A

**Table A1:** Management of Dabrafenib and Trametinib-related Pyrexia in Canadian Routine Practice: Preliminary Survey of Canadian Centres (N = 12).

How do you manage the first occurrence of pyrexia?			
Treatment interruption of both drugs	Treatment interruption of dabrafenib only	Acetaminophen and ibuprofen as needed only	
66%	25%	8%	
When do you resume treatment?			
24 h post-resolution of symptoms	48 h post-resolution of symptoms		
50%	50%		
At what point do you consider steroids?			
After 2nd occurrence	After ≥2 occurrences	When refractory to dose reduction	Other ^a^
8%	42%	42%	8%
At what point do you consider dose reduction?			
After 2nd occurrence	After ≥2 occurrences	When refractory to steroids	Other ^b^
25%	42%	25%	8%
At what point do you consider permanently discontinuing dabrafenib and trametinib?			
≥2 occurrences	Refractory to dose reduction	Other ^c^	
8%	42%	50%	

^a^ It depends on the severity of the first occurrence; if mild and short-lived, I might wait until 2 or more occurrences. ^b^ Refractory to medical treatment (e.g., steroid) and having other complications (increase in serum creatinine, electrolytes abnormalities, etc.). ^c^ Significant clinical event, at least grade 3; after all strategies fail; clinical signs or symptoms despite dose reduction(s); Refractory to dose reduction/steroids and other end-organ damage.

## References

[1] Long G., Flaherty K.T., Stroyakovskiy D., Gogas H., Levchenko E., de Braud F., Larkin J., Garbe C., Jouary T., Hauschild A. (2017). Dabrafenib plus trametinib versus dabrafenib monotherapy in patients with metastatic BRAF V600E/K-mutant melanoma: Long-term survival and safety analysis of a phase 3 study. Ann. Oncol..

[2] Robert C., Karaszewska B., Schachter J., Rutkowski P., Mackiewicz A., Stroiakovski D., Lichinitser M., Dummer R., Grange F., Mortier L. (2015). Improved Overall Survival in Melanoma with Combined Dabrafenib and Trametinib. N. Engl. J. Med..

[3] Larkin J., Ascierto P.A., Dréno B., Atkinson V., Liszkay G., Maio M., Mandalà M., Demidov L., Stroyakovskiy D., Thomas L. (2014). Combined Vemurafenib and Cobimetinib in BRAF-Mutated Melanoma. N. Engl. J. Med..

[4] Dummer R., Ascierto P.A., Gogas H.J., Arance A., Mandala M., Liszkay G., Garbe C., Schadendorf D., Krajsova I., Gutzmer R. (2018). Overall survival in patients with BRAF-mutant melanoma receiving encorafenib plus binimetinib versus vemurafenib or encorafenib (COLUMBUS): A multicentre, open-label, randomised, phase 3 trial. Lancet Oncol..

[5] Dummer R., Hauschild A., Santinami M., Atkinson V., Mandalà M., Kirkwood J.M., Chiarion Sileni V., Larkin J., Nyakas M., Dutriaux C. (2020). Five-Year Analysis of Adjuvant Dabrafenib plus Trametinib in Stage III Melanoma. N. Engl. J. Med..

[6] Daud A., Tsai K. (2017). Management of Treatment-Related Adverse Events with Agents Targeting the MAPK Pathway in Patients with Metastatic Melanoma. Oncologist.

[7] Menzies A.M., Ashworth M.T., Swann S., Kefford R., Flaherty K., Weber J., Infante J.R., Kim K.B., Gonzalez R., Hamid O. (2015). Characteristics of pyrexia in BRAFV600E/K metastatic melanoma patients treated with combined dabrafenib and trametinib in a phase I/II clinical trial. Ann. Oncol..

[8] Lee C.I., Menzies A.M., Haydu L.E., Azer M., Clements A., Kefford R.F., Long G.V. (2014). Features and management of pyrexia with combined dabrafenib and trametinib in metastatic melanoma. Melanoma Res..

[9] Long G.V., Stroyakovskiy D., Gogas H., Levchenko E., De Braud F., Larkin J., Garbe C., Jouary T., Hauschild A., Grob J.J. (2014). Combined BRAF and MEK Inhibition versus BRAF Inhibition Alone in Melanoma. N. Engl. J. Med..

[10] Weber J., Del Vecchio M., Mandala M., Gogas H., Arance A., Dalle S., Cowey C., Schenker M., Grob J., Chiarion-Sileni V. (2019). Analysis of pyrexia in patients (pts) treated with dabrafenib (D) and/or trametinib (T) across clinical trials. Ann. Oncol..

[11] Schadendorf D., Hauschild A., Santinami M., Atkinson V., Mandalà M., Sileni V.C., Larkin J., Nyakas M., Dutriaux C., Haydon A. (2019). Patient-reported outcomes in patients with resected, high-risk melanoma with BRAFV600E or BRAFV600K mutations treated with adjuvant dabrafenib plus trametinib (COMBI-AD): A randomised, placebo-controlled, phase 3 trial. Lancet Oncol..

[12] Ascierto P.A., McArthur G.A., Dréno B., Atkinson V., Liszkay G., Di Giacomo M., Mandalà M., Demidov L., Stroyakovskiy D., Thomas P.L. (2016). Cobimetinib combined with vemurafenib in advanced BRAF(V600)-mutant melanoma (coBRIM): Updated efficacy results from a randomised, double-blind, phase 3 trial. Lancet Oncol..

[13] Dummer R., Ascierto P.A., Gogas H.J., Arance A., Mandala M., Liszkay G., Garbe C., Schadendorf D., Krajsova I., Gutzmer R. (2018). Encorafenib plus binimetinib versus vemurafenib or encorafenib in patients with BRAF -mutant melanoma (COLUMBUS): A multicentre, open-label, randomised phase 3 trial. Lancet Oncol..

[14] Flaherty K.T., Robert C., Hersey P., Nathan P., Garbe C., Milhem M., Demidov L.V., Hassel J.C., Rutkowski P., Mohr P. (2012). Improved Survival with MEK Inhibition in BRAF-Mutated Melanoma. N. Engl. J. Med..

[15] Infante J.R., Fecher L.A., Falchook G.S., Nallapareddy S., Gordon M.S., Becerra C., De Marini D.J., Cox D.S., Xu Y., Morris S.R. (2012). Safety, pharmacokinetic, pharmacodynamic, and efficacy data for the oral MEK inhibitortrametinib: A phase 1 dose-escalation trial. Lancet Oncol..

[16] Ouellet D., Gibiansky E., Leonowens C., O’Hagan A., Haney P., Switzky J., Goodman V.L. (2014). Population pharmacokinetics of dabrafenib, a BRAF inhibitor: Effect of dose, time, covariates, and relationship with its metabolites. J. Clin. Pharmacol..

[17] Hajek E., Krebs F., Bent R., Haas K., Bast A., Steinmetz I., Tuettenberg A., Grabbe S., Bros M. (2018). BRAF inhibitors stimulate inflammasome activation and interleukin 1 beta production in dendritic cells. Oncotarget.

[18] Atkinson V., Long G., Menzies A., McArthur G., Carlino M.S., Millward M., Roberts-Thomson R., Brady B., Kefford R., Haydon A. (2016). Optimizing combination dabrafenib and trametinib therapy in BRAF mutation-positive advanced melanoma patients: Guidelines from Australian melanoma medical oncologists. Asia-Pac. J. Clin. Oncol..

[19] (2021). MEKINIST Product Monograph, Novartis Pharmaceuticals Canada Inc.. https://www.ask.novartispharma.ca/download.htm?res=mekinist_scrip_e.pdf&resTitleId=1087.

[20] (2021). TAFINLAR Product Monograph, Novartis Pharmaceuticals Canada Inc.. https://www.ask.novartispharma.ca/download.htm?res=tafinlar_scrip_e.pdf&resTitleId=1095.

[21] Long G.V., Hauschild A., Santinami M., Atkinson V., Mandalà M., Chiarion-Sileni V., Larkin J., Nyakas M., Dutriaux C., Haydon A. (2017). Adjuvant Dabrafenib plus Trametinib in Stage III BRAF-Mutated Melanoma. N. Engl. J. Med..

[22] A Study of the Anti-PD1 Antibody PDR001, in Combination with Dabrafenib and Trametinib in Advanced Melanoma (COMBI-i). https://clinicaltrials.gov/ct2/show/NCT02967692.

[23] Study of Dabrafenib+ Trametinib in the Adjuvant Treatment of Stage III BRAF V600+ Melanoma after Complete Resection to Evaluate the Impact on Pyrexia Related Outcomes (COMBI-APlus). https://clinicaltrials.gov/ct2/show/NCT03551626.

[24] Walter E.J., Hanna-Jumma S., Carraretto M., Forni L. (2016). The pathophysiological basis and consequences of fever. Crit. Care.

[25] Loblaw D.A., Prestrud A.A., Somerfield M.R., Oliver T.K., Brouwers M.C., Nam R.K., Lyman G.H., Basch E. (2012). American Society of Clinical Oncology Clinical Practice Guidelines: Formal Systematic Review–Based Consensus Methodology. J. Clin. Oncol..

[26] Falkson C., Bezjak A., Darling G., Gregg R., Malthaner R., Maziak D.E., Yu E., Smith C.A., McNair S., Ung Y.C. (2009). The Management of Thymoma: A Systematic Review and Practice Guideline. J. Thorac. Oncol..

[27] Algazi A. Continuous dosing of BRAF and MEK inhibitors improves PFS in melanoma subset Abstract CT02-01. Proceedings of the AACR Annual Meeting.

[28] Tkinson V., Robert C., Grob J.J., Gogas H., Dutriaux C., Demidov L.V., Gupta A., Menzies A.M., Ryll B., Miranda F. (2021). Improved pyrexia-related outcomes associated with an adapted pyrexia adverse event (AE) management algorithm in patients (pts) treated with adjuvant dabrafenib + trametinib (dab + tram): Primary results of COMBI-APlus. ASCO.

[29] Ascierto P.A., Robert C., Nathan P.D., Dummer R., Tawbi H.A.-H., Flaherty K.T., Ribas A., Schadendorf D., Green S., Sandalic L. (2021). Pyrexia-related outcomes upon application of an adapted pyrexia management algorithm in patients (pts) with BRAF V600: Mutant unresectable or metastatic melanoma treated with dabrafenib plus trametinib (DabTram) in the COMBI-i trial. J. Clin. Oncol..

